# Nature’s Own Pharmacy: Mushroom-Based Chemical Scaffolds and Their Therapeutic Implications

**DOI:** 10.3390/ijms242115596

**Published:** 2023-10-26

**Authors:** Mubashir Hassan, Saba Shahzadi, Richard F. Ransom, Andrzej Kloczkowski

**Affiliations:** 1 The Steve and Cindy Rasmussen Institute for Genomic Medicine, Nationwide Children’s Hospital, Columbus, OH 43205, USA; mubasher.hassan@nationwidechildrens.org (M.H.); saba.shahzadi@nationwidechildrens.org (S.S.); 2 Funite LLC., Ann Arbor, MI 48104, USA; artist@livedye.com; 3 Department of Pediatrics, The Ohio State University, Columbus, OH 43205, USA

**Keywords:** mushroom, metabolites, medicine, β-glucan, terpenoids

## Abstract

Mushrooms are new potential sources of valuable medicines, long neglected because of difficulties experienced in their cultivation. There is a large variety of medicinal mushrooms which possess significant therapeutic properties and are used as medications for various diseases because they contain several novel highly bioactive components. Medicinal mushrooms can be identified based on their morphology, size, mass, and the color of the stalk, cap and spore, and attachment to the stalk. Medicinal mushrooms possess a variety of important biological activities and are used as antioxidants, hepatoprotectors, anticancer, antidiabetic, anti-inflammatory, antiaging, antiviral, antiparasitic, and antimicrobial agents, among others. This review provides a basic overview of the chemical scaffolds present in mushrooms and their therapeutic implications in the human body.

## 1. Introduction

Mushrooms are the fleshy, spore-bearing fruiting bodies of specific species of the fungi kingdom, typically growing above ground on soil. The existence of fungi has been observed in all climatic zones, primarily on land, but also in water (fresh and salt). By 2020, approximately 148,000 species of mushrooms had been discovered, but the actual number is much higher. About 2000 new species are discovered every year. About 90% of fungi remain unknown. Using data obtained from areas where most living organisms have been identified, it is estimated that there are approximately 1–1.5 million species of fungi (four times more than seed plants). The number of known mushroom species also changes due to changes in their taxonomy [1]. Humans have long been ingesting mushrooms for both their pleasing flavor and nutritional values along with their medicinal properties [2,3,4]. Mushrooms are a significant source of essential nutrients including carbohydrates, dietary fibers, vitamins (B1, B2, B12, C, D, and E), minerals, and proteins [5,6]. Mushrooms produce bioactive molecules and compounds such as polysaccharides [7], lectins [8], phenolic compounds [9], and tocopherols (methylated phenols), as well as other complex compounds [10]. More precisely, multiple bioactive compounds including volatile oils (*Ganoderma pfeifferi* [11]), flavonoids (*Chaetomium globosum* and *Xylariaceae* [12,13]), alkaloids (psychedelic mushrooms [14]), fats, polysaccharides (*Ganoderma lucidum, Trametes versicolor* and *Grifola frondose* [15]), tocopherols (*Paxillus involutus* and *Pisolithus arhizus* [16]), glycosides, carotenoids, terpenoids, lectins, enzymes, phenolics, and folates are present in mushroom species [17]. 

## 2. Mushrooms with Medicinal Traits

### 2.1. Coriolus versicolor (C. versicolor)

**Figure 1:**
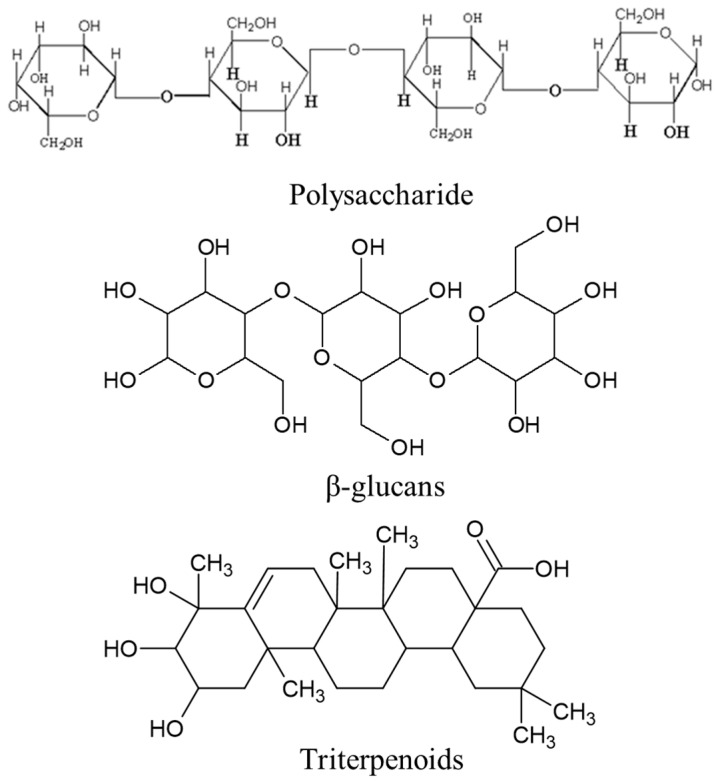
Bioactive compounds of *C. versicolor*.

*C. versicolor* is a mushroom that belongs to the *Polyporaceae* family [18]. *C. versicolor* has a distinctive fan-like or turkey tail-shaped fruiting body, with concentric rings of different colors. *C. versicolor* has a long history of use in traditional Chinese medicine and other traditional healing systems [19]. The *C. versicolor* contains various bioactive compounds, including polysaccharides, β-glucans, and triterpenoids (Figure 1), which are believed to contribute to its medicinal properties [20]. β-glucan is used to repair the activities of pancreatic tissues by increasing the production of insulin from β-cells, and by decreasing the levels of glucose in the blood [21]. It is commonly used as an immunomodulator and has been studied for its potential anticancer, antiviral, and anti-inflammatory effects. Prior studies showed that polysaccharides and β-glucans found in *C. versicolor* could stimulate the immune system, enhancing its ability to fight infections and diseases [22,23,24]. Several studies showed that extracts from *C. versicolor* exhibited anticancer effects, including inhibiting the growth of certain cancer cells and enhancing the activity of the immune system against cancer [25]. Other research showed that *C. versicolor* extracts may have antiviral properties and could be effective against certain viral infections, including the human papillomavirus (HPV) and hepatitis C virus (HCV) [26]. *C. versicolor* is commonly included in dietary supplements in the form of capsules, powders, and extracts [27]. *Tinea versicolor* (*T. versicolor*) contains a compound called polysaccharide-K (PSK), which has been investigated for its potential anticancer properties and immune-modulating effects [28]. PSK has been shown to have various effects on the immune system, including stimulating the production and activity of immune cells such as natural killer cells, T cells, and macrophages [29,30]. PSK has been extensively studied for its potential anticancer properties and is commonly used as an adjuvant therapy in tumor treatment, particularly in Japan and other Asian countries [31,32].

### 2.2. Agaricus campestris (A. campestris) 

Agaricus species have been used to treat heart and cardiovascular disorders, arteriosclerosis, stomach ulcers, tumors, liver disease and digestive problems, high cholesterol, type 2 diabetes, and other medical problems [17,33]. Of these, *A. campestris* is a widely consumed gilled mushroom closely related to the button mushroom *Agaricus bisporus* [34]. It is often found in fields or meadows. *A. campestris* has been studied for its potential health benefits and has been consumed to boost the immune system and to cope with physical and emotional stress. *A. campestris* has been noted for its insulin-releasing and glucose level-lowering activities [35]. Lectins isolated from the *A. campestris* have been found to increase the insulin released by isolated rat islets of Langerhans. It has also been used in the treatment of diabetes [36,37].

### 2.3. Ganoderma lucidum (G. lucidum)

*G. lucidum* is a type of medicinal mushroom that has been highly valued in traditional Chinese medicine [38]. *G. lucidum* is known for its distinctive kidney-shaped cap and woody texture [39]. *G. lucidum* is usually reddish-brown in color, has a bitter taste, and has been used in the form of extracts, powders, or capsules rather than being eaten directly [40]. *G. lucidum* is considered a powerful adaptogen, which means it helps the body adapt to various stressors and restore balance. It is believed to support overall health and longevity and has been used to enhance the immune system, promote liver health, reduce inflammation, and improve sleep quality [41,42]. *G. lucidum* contains bioactive compounds such as triterpenoids, polysaccharides, and peptidoglycans, which are thought to contribute to its medicinal properties [43]. Some studies suggest that it may have antioxidant, anti-inflammatory, and anticancer effects. *G. lucidum* may have an antitumor effect, achieved by boosting the host’s immune function [44,45]. Another study reported that *G. lucidum* polysaccharides may be involved in boosting cytokine production, dendritic cell maturation and the functioning of cytotoxic T lymphocytes, and cytokine-induced killer cells (CIK) [46,47]. 

Triterpenes present in *G. lucidum* [48] prevent metastatic growth by regulating interleukin-8 (IL-8) and matrix metalloproteinase-9 (MMP-9), and in macrophage cells they suppress the production and release of inflammatory cytokines. Triterpenes induce cell cycle arrest by downregulating cyclin D1 at the G1 phase, and by lowering the activity of protein kinase C (PKC) at the G2 phase and promoting tumor cell apoptosis via mitochondria-dependent pathways by activating the caspase cascade [49,50,51]. The chemical constituents derived from the fruiting bodies of *G. lucidum* have been thoroughly investigated, as well as their bioactivities. Triterpenoids are the most significant active substances in numerous pharmacological applications [52,53]. Several triterpenoids have been shown to have antiangiogenic properties, meaning they can inhibit the formation of the new blood vessels necessary for the growth and metastasis of tumors [54]. For example, compounds such as betulinic acid, ursolic acid, and oleanolic acid have been shown to inhibit angiogenesis by targeting various molecular pathways involved in this process [55]. 

### 2.4. Hydnum repandum (H. repandum)

*H. repandum* is commonly known as the hedgehog mushroom because of its distinctive pore-producing structures. It is often found in moderate regions of Europe, Asia, and North America. The fruiting body of *H. repandum* is distinctive and easily recognizable due to its convex to funnel-shaped cap with a wavy margin. *H. repandum* is highly regarded as an edible mushroom and is sought after by mushroom enthusiasts and foragers. *H. repandum* has exhibited strong antioxidant activity and is considered a significant source of dietary nutrients [56].

**Figure 2:**
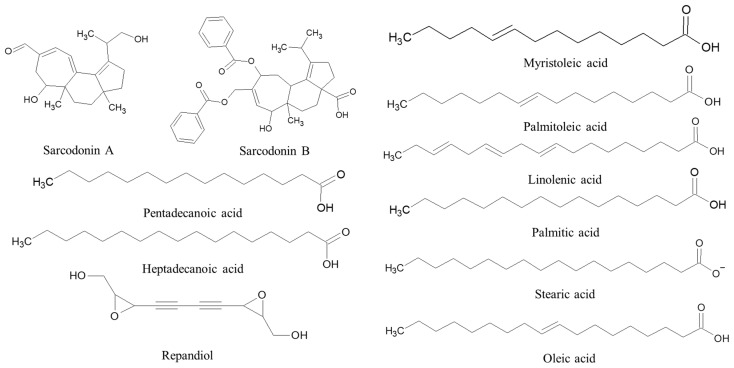
Bioactive compounds extracted from *H. repandum*.

Repandiol is a compound produced by H. repandum that demonstrates cytotoxic activity against various cancer cell types, primarily colon adenocarcinoma cells. Chloroform extracts from this mushroom have a mildly antibiotic effect against *Staphylococcus epidermidis*, *Staphylococcus aureus, Enterobacter aerogenes*, and *Bacillus subtilis.* Ethanol extracts are active only against *Bacillus subtilis* [17,56]. Furthermore, sarcodonin A and scabronine B, along with other related compounds, have been isolated from *H. repandum* [57]. Fatty acids like oleic, stearic, pentadecanoic, heptadecanoic, palmitoleic, linolenic, palmitic and myristoleic acids were found in the fruiting bodies of *H. repandum* (Figure 2) [58]. In another study, it was observed that *H. repandum* exhibited good antioxidant, proapoptotic, cytotoxic, and antiproliferative activities [59]. The impact of extracts from *H. repandum* on the development and sporulation of *Penicillium expansum* was examined and generated results showing substantial inhibition of mycelial growth and a reduction in pathogen sporulation [60]. 

### 2.5. Coprinus plicatilis (C. plicatilis)

**Figure 3:**
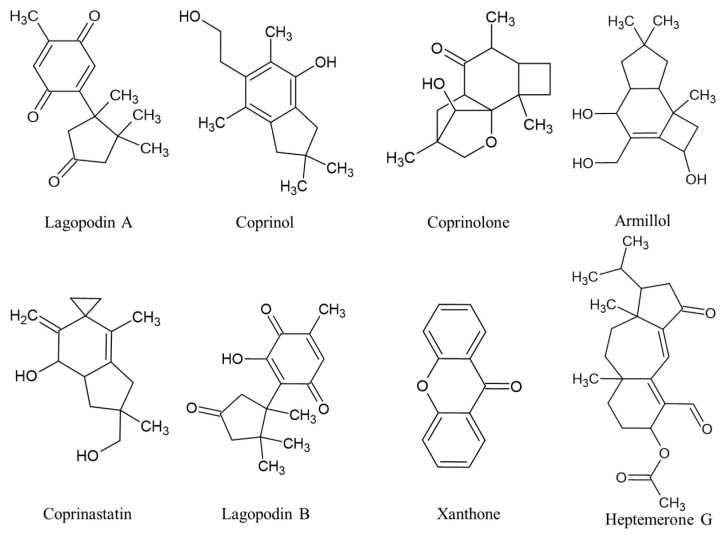
Bioactive compounds extracted from *C. plicatilis*.

*C. plicatilis* (or *Parasola plicatilis*), commonly known as the “shaggy ink cap”, is a species of mushroom belonging to the genus *Coprinus* [61]. It is a basidiomycete fungus found in many parts of the world, typically growing in grassy areas, lawns, meadows, and woodlands. The cap of *C. plicatilis* starts out convex and expands up to 2–6 cm diameter. *C. plicatilis* is generally considered edible when consumed in its early stages before the cap begins to blacken and liquefy [62]. The Coprinus sp. are rich in secondary metabolites such as terpenoids, including sesquiterpenes such as the lagopodins [63], coprinol [64], coprinolone [65], illudins [66], armillol, coprinastatin, heptemerones, coprinacins, and xanthothone, respectively [67] (Figure 3). These compounds exhibit a wide spectrum of biological activities, ranging from antibacterial, cytotoxic, antifungal to nematicidal [67].

### 2.6. Morchella vulgaris (M. vulgaris) 

**Figure 4:**
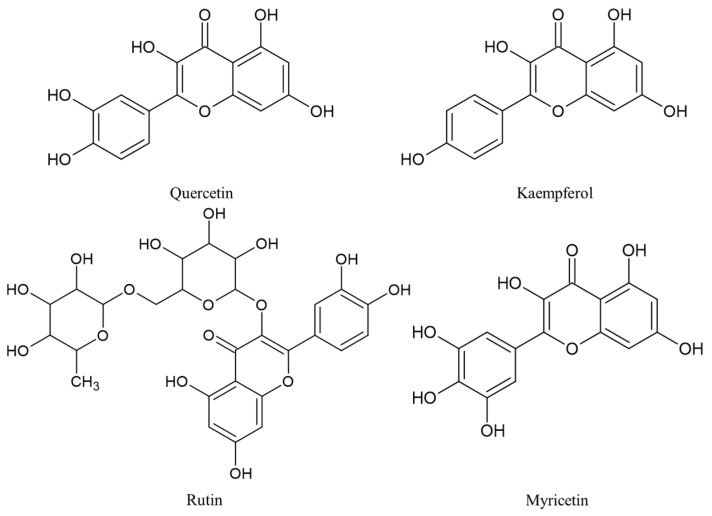
Bioactive compounds extracted from *M. vulgaris*.

*M. vulgaris*, commonly known as the common morel, is a species of edible mushroom [68]. Phylogenetic and nomenclatural studies confirmed the status of *M. vulgaris* as a distinct species and resolved several of its synonymities [69]. The common morels have a unique and recognizable appearance with honeycomb-like caps with ridges and pits that give them a sponge-like texture. The caps can vary in color from pale tan to dark brown and are usually conical or cylindrical in shape [70,71,72]. *M. vulgaris* and other morel species have been studied for their bioactive compounds. There are some bioactive compounds found in morels which include antioxidants, polysaccharides, vitamin D, amino acids, and terpenes [72,73]. Morels are rich in antioxidants, which help protect the body against oxidative stress caused by free radicals. These antioxidants include compounds such as phenolic compounds, flavonoids (including quercetin, rutin, kaempferol, and myricetin), and ascorbic acid (vitamin C). Morels also contain various polysaccharides which are complex carbohydrates that possess immunomodulating, anti-inflammatory, and antitumor properties [74]. *M. vulgaris* is also a natural source of vitamin D which is essential for bone health, immune function, and various other physiological processes in the body. Morels contain several essential amino acids, including lysine, leucine, and valine. Morels also contain various terpenes, which are organic compounds responsible for the characteristic aroma and flavor of the mushroom [75]. Some terpenes found in morels, such as sesquiterpenes, have been investigated for their potential anti-inflammatory and anticancer activities. The selected compounds are depicted in Figure 4. 

### 2.7. Cantherullus cibarius (C. cibarius)

*C. cibarius*, frequently known as the chanterelle mushroom, is a species of edible fungi that belongs to the Cantharellus genus [76]. It is highly regarded in culinary circles for its distinctive flavor and texture. While chanterelles are primarily appreciated for their culinary uses, they also contain bioactive compounds that have been of interest to researchers. *C. cibarius* produces polysaccharides that have been shown to have a variety of biological activities, including immunomodulatory, antioxidant, and anti-inflammatory effects [77]. Polysaccharides extracted from *C. cibarius* possess immunomodulatory properties, which may help enhance immune response [78]. Furthermore, a polysaccharide fraction from *C. cibarius* can prevent the activity of both COX-1 and COX-2 and inhibit colon cell proliferation without significant toxicity towards normal cells. Moreover, *C. cibarius* produces a water-soluble polysaccharide that has potential immune-stimulating activity applications as a nutraceutical product [79]. *C. cibarius* also contains terpenoids with antioxidant and antimicrobial properties [80]. Furthermore, *C. cibarius* contains significant amounts of vitamins, particularly vitamin D, which plays a significant role in calcium absorption, bone health, and immune function [81]. 

### 2.8. Amanita muscaria (A. muscaria)

**Figure 5:**
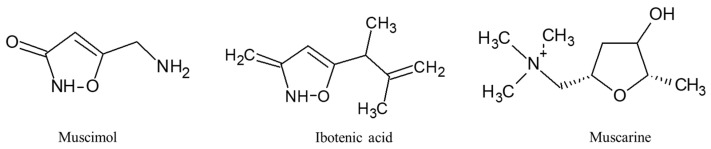
Bioactive compounds extracted from *A. muscaria*.

*A. muscaria,* generally known as the fly agaric or fly amanita, is a basidiomycete of the genus *Amanita* [82]. *A. muscaria* is widely recognized for its distinctive appearance with a bright red cap and white spots, remnants of the universal veil that covers the entire emerging mushroom during development [82]. *A. muscaria* is present throughout the temperate and boreal regions of the northern half of the Earth and in many countries in the southern half, commonly in symbiosis with pine and birch trees. *A. muscaria* contains several bioactive compounds that can have various effects on the human body (Figure 5). However, it is important to note that *A. muscaria* is a toxic mushroom and ingesting it can be extremely dangerous or even fatal. The main bioactive compounds found in *A. muscaria* include muscimol, ibotenic acid and muscarine. Muscimol (3-hydroxy-5-aminomethyl-1-isoxazole) is the primary psychoactive compound in *A. muscaria*. It acts as a GABA receptor agonist, which means it enhances the inhibitory neurotransmitter activity in the brain. Muscimol binds to the same site on the GABAA receptor complex as GABA itself, in opposition to other GABAergic drugs (like benzodiazepines or barbiturates) which bind to distinct regulatory sites [83]. GABAA receptors are commonly occurring in the brain, so when muscimol is administered, it modifies neuronal activities in multiple brain regions such as the cerebellum, cerebral cortex, and hippocampus [84,85]. Ibotenic acid ((S)-2-amino-2-(3-hydroxyisoxazol-5-yl)acetic acid) is a prodrug of muscimol, meaning it is converted into muscimol in the body. It has similar psychoactive effects to muscimol, but it is also known to cause excitotoxicity, which can damage nerve cells in the brain. Ibotenic acid is a potent agonist of the NMDA and group I and II metabotropic glutamate receptors, and a weak agonist of the AMPA and kainate receptors [86]. Muscarine is not the primary psychoactive compound in *A. muscaria* [82]. It is a toxin that primarily acts as a cholinergic agonist, stimulating the parasympathetic nervous system [87]. Muscarine can cause symptoms such as increased salivation, sweating, tearing, and blurred vision. *A. muscaria* should not be consumed recreationally or for any other purpose due to its high toxicity. The effects of ingestion can be unpredictable, and severe poisoning can occur, leading to symptoms such as nausea, vomiting, diarrhea, confusion, seizures, and even coma [88].

### 2.9. Cortinarius violaceus (C. violaceus)

**Figure 6:**
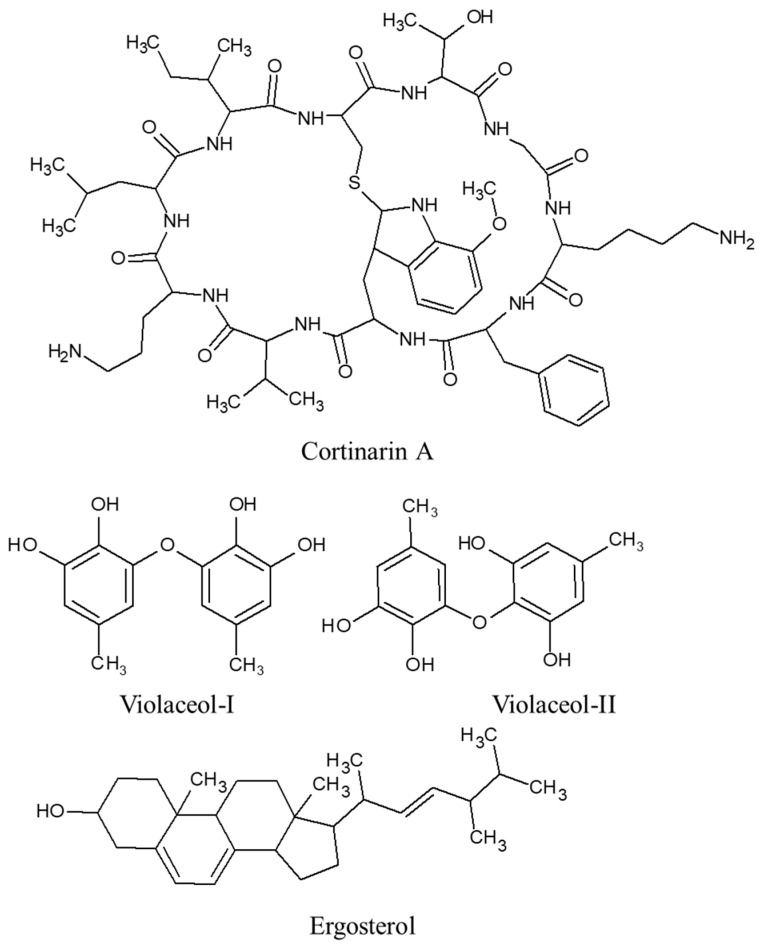
Bioactive compounds extracted from *C. violaceus*.

*C. violaceus*, frequently known as the violet webcap, is a species of mushroom belonging to the *Cortinarius* genus [89]. It is known for its vibrant purple color and is found in forests across Europe and North America [90]. *C. violaceus* has been studied for its chemical compositio and multiple bioactive compounds have been identified in this mushroom, such as cortinarin A, cortiloxin A, violaceol-I and -II, and ergosterol (Figure 6). Cortinarin A has been studied for its antimicrobial activity against various bacteria and fungi. It has also shown potential anti-inflammatory properties [91,92]. Violaceol-I and Violaceol-II are bioactive compounds that have been found to inhibit the growth of certain fungi [93,94]. Similarly, ergosterol is a sterol compound found in *C. violaceus* which has been associated with various biological activities, including antifungal, anti-inflammatory, and antioxidant properties [95]. However, due to the potential toxicity of *C. violaceus*, direct consumption of this mushroom is to be avoided.

### 2.10. Tremella mesenterica (T. mesenterica)

*T. mesenterica*, generally known as “witches’ butter” or “yellow brain”, is a species of jelly fungus found in many parts of the world [96]. It is characterized by its gelatinous and rubbery texture as well as its bright yellow or orange color. *T. mesenterica* is a saprophytic fungus, meaning it obtains nutrients from decomposing dead organic matter, particularly wood [97]. *T. mesenterica* contains several bioactive compounds that may have potential health benefits. There are reports that *Tremella* species produce polysaccharides which may be effective in cancer prevention and immune system enhancement [98]. The polysaccharide, known as glucuronoxylomannan, is produced by the mushroom’s fruiting bodies as well as in mycelial culture, and consists of a mannan backbone glycosylated with xylan side chains in a regular repeating structure [96]. Studies have shown that glucuronoxylomannan from *T. mesenterica* has antiallergic, immunostimulatory, antidiabetic, anti-inflammatory, hepatoprotective, and hypocholesterolemic effects [99,100]. *T. mesenterica* is also a natural source of mannitol (C6H14O6), a sugar alcohol. Mannitol has diuretic properties and is sometimes used as a natural sweetener. It may have possible applications in the management of certain medical conditions, such as glaucoma and cerebral edema [101].

### 2.11. Rigidoporus microporus (R. microporus) 

*R. microporus* is a species of wood-decaying fungus belonging to the family Meripilaceae. It is commonly known as the white rot fungus or the rubbery agaric. This fungus is widely distributed in tropical and subtropical regions and is widely known for its ability to break down lignin, a complex polymer found in plant cell walls [102]. *R. microporus* produces polysaccharides shown to have immunomodulatory effects, including enhancing the production of immune cells and increasing the activation of natural killer cells and macrophages, which are important components of the immune response [17]. Furthermore, terpenoids and phenolic compounds have been extracted that may have activity against various bacteria as well as possible therapeutic activities in neurodegenerative disorders [103,104]. 

### 2.12. Grifola frondosa (G. frondosa) 

*G. frondosa* is a Basidiomycetes fungus belonging to the family of *Grifolaceae* and the order of *Polyporales*. The northeastern part of Japan, as well as the mild forests of eastern North America, Europe and Asia form the best environment for its occurrence. It is a very common mushroom in North America, called sheep’s head, king of mushrooms, hen-of-the-woods, or cloud mushroom [105]. *G. frondosa* is edible and viewed as a healthy nutrient, being a good source of fiber, protein, carbohydrate [106,107], vitamins (like D2) [108] and various minerals (such as K, P, Na, Ca, Mg) [106]. In addition to its high nutritional value, *G. fondosa* possesses various pharmacological properties [109]. Many bioactive polysaccharide fractions (like the MD, X, MZ, Grifolan, and MT-α-glucan fraction) have been obtained from *G. frondosa* and reported to have various bioactive properties, such as immunomodulation, antitumor, antivirus, antidiabetic, and anti-inflammatory agents [109]. Furthermore, glycoproteins from *G. frondose* have been reported to have antitumor [110], immune-enhancing [111], antidiabetic, antihypertensive, antihyperlipidemic, and antiviral effects [112]. Additionally, various small biomolecular compounds in *G. frondosa* possess anti-inflammatory, antitumor, hypoglycemic, and antioxidation properties [109]. 

### 2.13. Cordyceps sinensis (C. sinensis)

*C. sinensis*, known as the caterpillar fungus, is an entomopathogenic fungus. It mainly grows in the meadows above 3500 m in Tibet and Himalaya, mostly in Bhutan and Nepal [113,114]. Caterpillar fungus contains cordycepin, an adenosine derivative that has been studied for its antiviral and anticancer properties [115]. Cordycepin has shown inhibitory effects on DNA and RNA synthesis, making it a potential inhibitor of various cellular processes. Furthermore, it has also exhibited good antimicrobial, antiviral, anti-inflammatory, and anticancer properties [116]. Cordycepin has also been investigated for its potential to treat viral infections, such as herpes, influenza, and hepatitis [117]. Furthermore, cordycepin has been found to possess anti-inflammatory properties, which may make it beneficial for conditions related to inflammation, such as asthma, arthritis, and certain skin disorders [118]. 

### 2.14. Hericium erinaceus (H. erinaceus)

**Figure 7:**
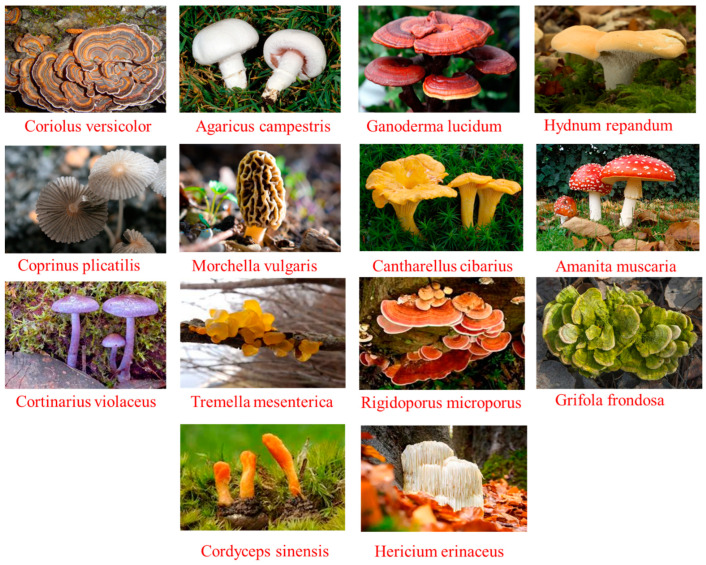
Varieties of mushrooms with medicinal significance.

*H. erinaceus*, also known as lion’s mane mushroom, is an edible mushroom belonging to the tooth fungus group [119,120]. *H. erinaceus* contains hericenones and erinacines, compounds shown to have potential neuroprotective effects [121,122]. Hericenones stimulate the production of nerve growth factors, such as brain-derived neurotrophic factor (BDNF), which is crucial for the growth and maintenance of neurons [123]. By promoting the growth and protection of neurons, hericenones may help to support brain health and potentially slow down the progression of neurodegenerative diseases such as Alzheimer’s [124]. Erinacines, on the other hand, are a group of compounds that stimulate the production of nerve growth factors and promote the formation of new neurons [125]. A clump of lion’s mane mushrooms is shown in Figure 7.

### 2.15. Stereochemistry of Biologically Active Compounds Extracted from Mushrooms 

**Figure 8:**
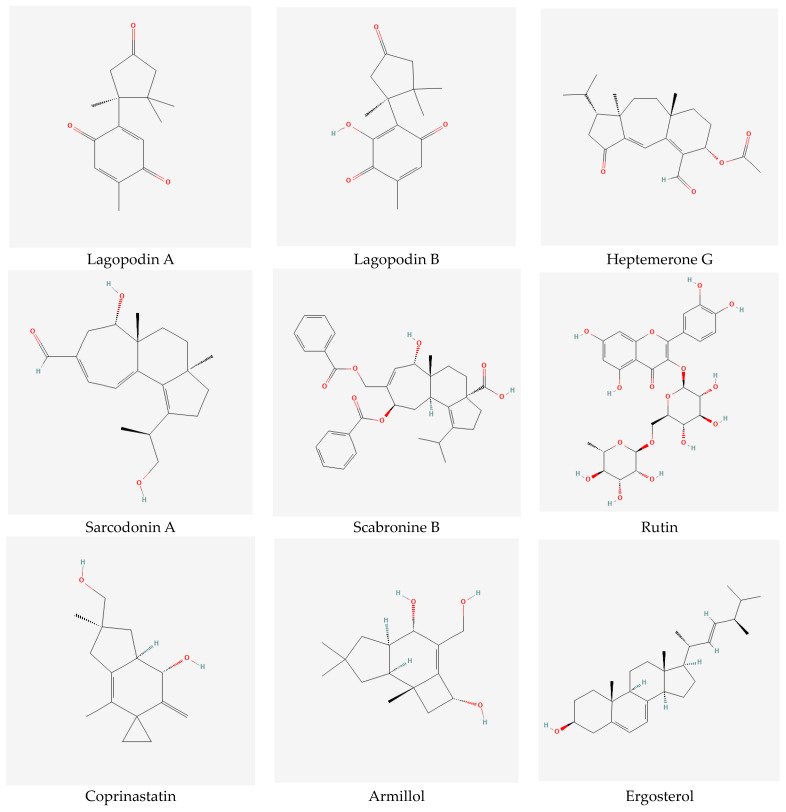
Chirality of biologically active stereoisomers of lagopodin A, lagopodin B, heptemerone G, sarcodonin A, scabronine B, rutin, coprinastatin, armillol, and ergosterol. All images were taken from PubChem, an open chemistry database at the National Institutes of Health.

Stereochemistry plays a very important role in most biologically active compounds, including those extracted from mushrooms. Frequently, molecules that have the same molecular formula and the sequence of bonds differ in the orientations of some molecular fragments in three-dimensional space. This so-called stereoisomerism is extremely important in biology and medicine, since some stereoisomers are biologically active, while others are biologically neutral. Figure 8 shows the stereochemical structures of the biologically active stereoisomers of: lagopodin A, lagopodin B, heptemerone G, sarcodonin A, scabronine B, rutin, coprinastatin, armillol, and ergosterol. All images in Figure 8 were taken from PubChem, an open chemistry database at the National Institutes of Health.

### 2.16. Mushrooms as Medicinal Therapeutics

Geographically, mushrooms occur from the high arctic to tropical regions all over the world. Mushrooms grow in swamps, forests, grasslands, pasture, gardens, and lawns [17,126]. However, there are some species that grow on waste like garbage, compost, and sawdust [127,128]. Mushrooms are increasingly seen as a source for new antimicrobial, and antinematodal agents with a variety of biological activities, in particular secondary metabolites including benzoic acid derivatives, terpenes, steroids, quinolones, anthraquinones, etc., along with certain primary metabolites like proteins, peptides, or oxalic acid [129]. Mushroom-based anthraquinones with reported bioactivities such as dermocybin, emodin, dermorubin, dermolutin, dermoglaucin, physcion, and others with their chloro derivatives are commonly found. Anthraquinones are naturally existing phenolic compounds based on the 9,10-anthraquinone skeleton and widely used in industry [130]. Dermocybin is a natural anthraquinone isolated from *Dermocybe sanguinea* and used as a disperse dye for polyester. Emodin is a naturally occurring anthraquinone compound found in plants (rhubarb, cascara, senna, Aloe vera, Polygonum multiflorum, Polygonum cuspidatum) and fungi (Aspergillus, Pyrenochaeta, and Pestalotiopsis). It is a stimulant laxative used to treat constipation as well as a treatment for viral, bacterial, and bowel abnormalities [131,132]. Physcion is a naturally occurring anthraquinone with antifungal and antitumor properties. Physcion is a major component of Radix, the traditional Chinese medicament. It has a variety of pharmacological properties, such as hepatoprotective, antimicrobial, antiproliferative, anti-inflammatory, and laxative activity [133]. Physcion can be applied to develop antibiotics, and antifungal agents, and in cell biology studies [134]. 

It has been observed that *Agaricus bisporus* is the most highly cultivated species followed by *Flammulina* sp., *Lentinus* sp., and *Pleurotus* sp., respectively. Mushrooms also contain non-digestible carbohydrates like raffinose (C_18_H_32_O_16_), chitin (C_64_H_106_N_8_O_41_), oligosaccharides, β-glucans, and resistant starch [135,136]. However, another study showed that both digestible and non-digestible carbohydrates are present in the mushroom [137]. Studies have reported that some mushrooms like *Trametes versicolor*, *Schizophyllum commune*, *Flammulina velutipes*, *Ganoderma lucidum*, *Phellinus linteus*, *Lentinus edodes*, *Cordyceps sinensis*, *Inonotus obliquus*, and *Grifola frondosa* exhibit inhibitory effects on tumor growth and on the action of immunoceuticals, mainly by boosting the immune system of the host. This process involves the activation of NK cells, macrophages, dendritic cells, T cells, and the increased release of cytokines [138]. 

### 2.17. Primary Metabolites and Therapeutic Implications

#### 2.17.1. Carbohydrates

Various carbohydrates such as fructose, xylose, glucose, maltose, rhamnose, trehalose, arabinose, sucrose, fucose, mannose, and mannitol have been observed in mushrooms [139,140]. Polysaccharides extracted from mushrooms have acidic or neutral properties and show anticancer activity. It has been observed that β-glucans are the most common polysaccharides in mushrooms, forming 50% of the fungal cell wall mass. Functional studies reported that β-glucans are involved in the antioxidant, neuroprotective, anticancer, immunomodulating and anticholesterolemic activities of some edible mushrooms [141]. 

#### 2.17.2. Lipids

Edible mushrooms express polyunsaturated fatty acids (such as α-linolenic acid, eicosapentaenoic acid, docosahexaenoic acid, linoleic acid, arachidonic acid, and γ-linolenic acid) which decrease serum cholesterol levels [142,143]. Tocopherols are the natural antioxidants in the lipid fraction, which possess peroxyl radical scavenging activity. They destroy the free radicals that are produced in cells. These antioxidants protect cells against various degenerative and cardiovascular diseases, and cancer [144,145]. Linoleic acid is an essential fatty acid that decreases blood pressure, lowers triglyceride levels, and protects against arthritis and cardiovascular diseases [146,147].

#### 2.17.3. Proteins and Peptides

Proteins and peptides are the important biologically active compounds in mushrooms having various health benefits such as the inhibition of enzymes, increasing the absorption and digestion of nutritional components, and the modulation of immune functions [148]. Proteins and peptides in mushrooms that have pharmaceutical potential include ribonucleases, lectins, laccases, ribosome-inactivating proteins, and fungal immunomodulatory proteins [149]. Lectins are glycoproteins of a non-immune origin that bind cell surface carbohydrates and possess important antiviral, antitumor, antifungal, antibacterial, and immunomodulatory activities [150]. Fungal immunomodulatory proteins are used as adjuvants in cancer treatment due to their important role in suppressing the metastasis of tumor cells [151]. It has been shown that cordymin, an antifungal peptide (molecular mass 10,906 kDa) extracted from the mushrooms *Cordyceps militaris* and *Cordyceps sinensis,* possesses anti-inflammatory activity [152,153]. Xylose-specific lectins extracted from Xylaria hypoxylon with a molecular mass 28.8 kDa exhibits anticancer and antimitogenic properties [154]. 

#### 2.17.4. Mushroom-Based Chemical Scaffolds 

Mushrooms are a well-known natural source of bioactive compounds having potential therapeutic properties, including antioxidants, antimicrobial agents, and compounds with anticancer activity [21]. Lentinan (*Lentinula edodes*) is a polysaccharide that has been studied for its immunomodulatory effects and potential anticancer properties. Lentinan is often used as an adjunctive therapy alongside conventional cancer treatments, such as chemotherapy and radiation therapy [155]. Research suggests that lentinan has immunomodulatory effects, meaning it can regulate and enhance the immune system’s response to cancer cells. It can stimulate certain immune cells, such as natural killer cells and T cells, which play a crucial role in recognizing and eliminating cancer cells. Lentinan has also been found to increase the production of cytokines, which are chemical messengers that help coordinate the immune response [156]. Lentinan also has a direct association with Alzheimer’s disease (AD). It has been shown that lentinan improves memory and learning ability by improving antioxidant capacity and regulating the expression of the hippocampus c-fos protein in brains of AD rats [157]. Moreover, lentinan reduced amyloid-β plaque formation, which is one of the hallmarks of AD. Additionally, due to its antioxidant behavior, it may protect brain cells from damage [158,159].

Eritadenine (*Lentinula edodes*) has been shown to have cholesterol-lowering effects by inhibiting an enzyme involved in cholesterol synthesis. It is also believed to have potential benefits in preventing cardiovascular diseases. However, it is important to note that the research on eritadenine is still limited, and more studies are needed to fully understand its effects [160]. In another animal research paper, eritadenine was found to reduce cholesterol levels by inhibiting an enzyme called HMG-CoA reductase, which is involved in cholesterol synthesis. By inhibiting this enzyme, eritadenine may help lower total cholesterol, LDL cholesterol and triglyceride levels. In addition to its potential cholesterol-lowering effects, eritadenine has also been studied for its potential anticancer properties [161]. Previous research suggests that eritadenine may inhibit the growth of cancer cells and induce apoptosis (programmed cell death) in certain types of cancer, including colon and leukemia cells. Moreover, eritadenine has been investigated for its potential anti-inflammatory and immunomodulatory effects. It may have the ability to modulate the immune system and reduce inflammation, which could be beneficial in certain inflammatory diseases. However, more studies are required to validate these findings and understand the underlying mechanisms. 

While these potential mechanisms sound promising, it is important to note that most of the research into eritadenine’s effects on AD has been conducted in animal models or in vitro studies. There is a lack of large-scale clinical trials to evaluate its effectiveness and safety in humans specifically for AD [161]. Overall, while eritadenine may have potential health benefits and could play a role in AD, further research is needed to fully understand its mechanisms of action and its potential as a therapeutic agent. Moreover, various mushrooms contain compounds that possess enzyme-inhibitory properties, such as cholinesterase inhibitors. These compounds have been investigated for their potential in treating neurological disorders like AD. It is important to note that while mushroom-based inhibitors show promise, further research is needed to fully understand their mechanisms of action, potential side effects, and optimal therapeutic applications. It is always advisable to consult with a healthcare professional before considering any new treatments or supplements. The mushroom-based compounds have been depicted in Table 1.

Mushrooms can accumulate selenium from their substrates and transform it into organic selenium metabolites such as selenium polysaccharides, selenium proteins, and selenoamino acids. The most bioavailable forms of selenium are selenomethionine and selenocysteine, which are organic forms of selenium. It has been observed that mushrooms are a great source of selenium, and one cup of cooked shiitake mushrooms contains 36 mcg of selenium, which is 65% of the daily value. Portabellos, criminis, and white button mushrooms also provide between 35 and 50% of the daily value per cup [162,163].

## 3. Conclusions and Future Prospects

Mushrooms are the fruiting bodies of fungi that offer potential in a variety of therapeutic, nutraceutical, nutritional, and other applications. Mushrooms are well known and appreciated worldwide for their nutritional and medicinal values. Medicinal mushrooms have various bioactive components that have been studied but there are still many mushrooms with unknown metabolites offering many possibilities of significant discoveries relevant to human health in the future. Genomics, proteomics, and metabolomics should play a major role in the significant improvement of our knowledge of medicinal mushrooms in the near future.

## Figures and Tables

**Table 1:** Mushroom metabolites, mechanism, and their applications [17].

Medicinal Mushrooms	Bioactive Components	Treatment/Applications	Mechanism
***Trametes***	Carbohydrates, lipids, proteins, flavanols, bioflavonoids, iso-flavonoids, flavones, flavanone, ergosterol, β-sitosterol, stigmast-5-en-3-ol, hydroxy methylquinoline, sesquiterpene, coriolin and de-oxycoriolic acid	Antibacterial, anticancer, insecticidal, antioxidant, antiproliferative, anticoagulant, antifungal, antidiabetic, hepatoprotective, antiparasitic, antiviral, anti-inflammatory, upper respiratory, digestive, urinary tract infections and chronic hepatitis	Immunostimulant, activation of macrophage and natural killer cell cytotoxicity.
***Agaricus***	Fatty acids, phenolics, amino acids, sugar and polyols, organic acids, lectins, unsaturated fatty acids (linoleic and linolenic acids), sterols, phenolic, indole compounds and nutraceuticals	Liver disease, cancer, digestive problems, high cholesterol, type 2 diabetes, arteriosclerosis, bloodstream disorders, heart disease, osteoporosis and stomach ulcers	Inhibits cell proliferation of some cancer cell lines, antioxidant activities, anti-inflammatory
***Ganoderma***	Triterpenoids, polysaccharides, proteins and peptides, terpenoids, phenols, glycoproteins, triterpenes, amino acids (lysine and leucine), ganoderic acids, nucleotides and their derivatives, peptidoglycans and steroids	Diabetes, infections, cancer, immune-system disorders, hepatoprotection, bacteriostasis, bronchitis, gastric ulcer, hepatopathy, asthma, insomnia, chronic hepatitis, nephritis, arthritis, hypertension, weakness, fatigue, cough, anti-atherosclerosis, antioxidant, anti-HIV, nephroprotective, antitumor, antihepatotoxic, cardiovascular, respiratory properties. It also decreases the level of blood pressure, inhibition of platelet aggregation as well as blood cholesterol, anti-inflammatory, analgesic, chemo preventive, chemo and radio protective, sleep promoting, antibacterial, antiviral, hypolipidemic, antifibrotic, antiandrogenic, antiangiogenic, antiherpetic, radical-scavenging, antiaging, hypoglycemic, estrogenic activities.	Immunomodulator (interleukin–12 production), nitric oxide synthase activation
***Hydnum***	Polyphenolic compounds such as phenolic acids, flavonoids, hydroxybenzoic acids, lignans, tannins, stilbenes, oxidized polyphenols, ferulic acid, sarcodonin A, savronine B and quercetin	Antioxidant, antimicrobial, genotoxic, protective against chemotherapeutics, cytotoxic activity against a variety of tumor cells type, mainly colon adenocarcima cells	Synthesis of nerve growth factor
***Coprinus***	Carbohydrates, dietary fibers, proteins and phenolic compounds.	Regulates the blood glucose level, hypoglycemic and has antitumor, antioxidative, hypolipidemic, antibacterial as well as immunomodulation effects	Regulates antioxidative homeostasis
***Morchella***	Sugars, organic acids, flavonoids, triglycerides, free fatty acids and sterols	Anti-inflammatory as well as antitumor activity against both ascites as well as solid tumors using ethanolic extracts, high antioxidant activity	Immunomodulator, increases the cytotoxic effect
***Cantharellus***	Phenolic compounds, terpenes, steroids, lectins, polysaccharides, proteins, phenolic compounds, flavonoids, and tannins	Excellent antihyperglycemic, antioxidant, wound healing, antimicrobial, iron-chelation, cytotoxicity, antihypoxic, anti-inflammatory activities	Causes cytotoxicity against angiotensin-converting enzymes
***Amanita***	Ibotenic acid, muscazone and muscimol	Antitumor, pesticidal, cytotoxic, antioxidant, anticancer, antibacterial, acetylcholinesterase, esterolytic, antiviral, antilarvicidal, antifungal, anti-inflammatory properties	Induces cascade-dependent apoptosis
***Cortinarius***	Amino acids, orellanine	Antioxidant, antihyperglycemic, wound healing, antimicrobial, iron-chelation, cytotoxicity, antihypoxic, anti-inflammatory	Inhibits protein synthesis
***Tremella***	Fatty acids, proteins, enzymes, polysaccharides, phenols, flavonoid, dietary fiber and trace elements.	Fights cancer, combats obesity, anti-aging, lower cholesterol, protects nerves and is anti-inflammatory.	Enzyme inhibition
***Rigidoporus***	Anthraquinones, alkaloids (nitrogen-containing metabolites; psilocybin), tannins, saponins, phlobatannins, steroids, flavonoids, terpenoids and cardiac glycosides.	Mitogenic activity, antihepatitis B surface antigen effect, plasma clotting activity, activation of alternative pathway complement, tumor suppressive effects	Exact mechanism is unknown, antioxidant activities, anti-inflammatory
***Grifola***	Polysaccharide (glucans), sesquiterpenes, glycoproteins, etc.	Antitumor, anti-inflammation, immunomodulation, antivirus, antidiabetic, immunity enhancing, anti-hypertensive, antioxidation, non-alcoholic fatty liver disease, hyperlipidemia and hyperglycemia.	Immunomodulator
***Lentinus***	Phenolic compounds, polysaccharides, terpenoids, sterols and lipids	Fungal infection, bronchial inflammation, hyperlipidemia, hepatitis, cancer, depressed immune function, heart disease, infectious disease, flu and colds, environmental allergies, urinary inconsistencies, hypertension, diabetes	Inhibitory effect on interleukin-1β, tumor necrosis factor α
***Pleurotus***	Terpenoids, steroidal glycosidase, tannin	Anticholesterol, anticancer, antiviral, antidiabetic, antioxidant, eye health, antibacterial and antiarthritic.	Hypocholesterolemic, atherogenesis inhibition
***Calocybe***	Ascorbic acid, lipids, riboflavin, amino acids, pyridoxine, vitamins, biotin, low fat, nicotinic acid, proteins, minerals (arsenic, zinc, potassium, manganese, calcium, phosphorus, magnesium, iron and sodium), Fibers	Reduces the triglycerides and total plasma cholesterol level and consequently decreases the chance of cardiovascular, artery and atherosclerosis-related disorders, like neurodegenerative diseases, anticarcinogenesis, antiageing, antiobesity, cardiovascular disease, anti-infectious, prevents physical injury, antitumor, also helps to reduce the risk of breast cancer	Immunomodulator, immunogenerator
***Huitlacoche***	Contains anthocyanins and phenolic compounds which are phytochemicals, phytosterol, polyphenols, flavonoids, proteins, amino acids, glutamic acid, lysine, serine, aspartic acid, glycine, total carbohydrates, arabinose, mannose, galactose, xylose, glucitol, mannitol, glycerol, heteroglycans, dietary fiber and homoglycans	Antitumoral, antimutagenic, immunomodulating, antiatherogenic, hyperlipidemic, hypoglycemic, anti-inflammatory as well as various other health promoting activities	Exact mechanism is unknown, antioxidant activities.
***Cordyceps***	Alkaloids, amino acids, proteins, carbohydrates, flavonoids, phenols, gums, mucilages, saponins, cordycepic acid and cordycepin substances	Improved reproductive activity, blood sugar metabolism, effects of enhanced utilization of oxygen and production of ATP. This mushroom protects the organs from diseases of the kidney, liver and heart.	Immunomodulating effects, enhancement of neuromuscular activity, endurance-enhancing activity.
